# Functional inference by ProtoNet family tree: the uncharacterized proteome of Daphnia pulex

**DOI:** 10.1186/1471-2105-14-S3-S11

**Published:** 2013-02-28

**Authors:** Nadav Rappoport, Michal Linial

**Affiliations:** 1 School of Computer Science and Engineering, The Hebrew University of Jerusalem, Jerusalem, 91904, Israel; 2 The Sudarsky Center for Computational biology, Department of Biological Chemistry, Institute of Life Sciences, The Hebrew University of Jerusalem, Jerusalem, 91904 Israel

## Abstract

**Background:**

Daphnia pulex (Water flea) is the first fully sequenced crustacean genome. The crustaceans and insects have diverged from a common ancestor. It is a model organism for studying the molecular makeup for coping with the environmental challenges. In the complete proteome, there are 30,550 putative proteins. However, about 10,000 of them have no known homologues. Currently, the UniProtoKB reports on 95% of the Daphnia's proteins as putative and uncharacterized proteins.

**Results:**

We have applied ProtoNet, an unsupervised hierarchical protein clustering method that covers about 10 million sequences, for automatic annotation of the Daphnia's proteome. 98.7% (26,625) of the Daphnia full-length proteins were successfully mapped to 13,880 ProtoNet stable clusters, and only 1.3% remained unmapped. We compared the properties of the Daphnia's protein families with those of the mouse and the fruitfly proteomes. Functional annotations were successfully assigned for 86% of the proteins. Most proteins (61%) were mapped to only 2953 clusters that contain Daphnia's duplicated genes. We focused on the functionality of maximally amplified paralogs. Cuticle structure components and a variety of ion channels protein families were associated with a maximal level of gene amplification. We focused on gene amplification as a leading strategy of the Daphnia in coping with environmental toxicity.

**Conclusions:**

Automatic inference is achieved through mapping of sequences to the protein family tree of ProtoNet 6.0. Applying a careful inference protocol resulted in functional assignments for over 86% of the complete proteome. We conclude that the scaffold of ProtoNet can be used as an alignment-free protocol for large-scale annotation task of uncharacterized proteomes.

## Background

Daphnia pulex is a key player in the aquatic ecosystems and an important component in the food web. It is a model organism for studying environmental challenges including toxic conditions [1]. D. pulex is the first crustacean whose genome was sequenced [2]. The crustaceans and insects have diverged from a common ancestor. Nevertheless, they exhibit extraordinary levels of phenotypic diversity. There are 30,550 model proteins, 95% of them are named 'putative uncharacterized'. Over a third of the sequences lack homologues [2], and thus are considered novel genes. A detailed analysis on the evolutionary trends of Daphnia genome indicates that extensive gene duplication events occurred. Importantly, many of these duplicated genes are under purifying selection [2]. It was proposed that the amount of duplicated genes reflects the harsh living environments of the family Daphniidae. Specifically, genes that appear in tandem duplicated clusters are significantly over-represented in transcriptomes from extreme ecological conditions [2].

Comparative genomics approaches are useful for the discovery of functional elements from newly sequenced genomes [3]. Such methods were successfully used for complete sequenced Drosophilae (12 species) [4], and genomes from various yeast strains [5]. Daphnia is the only available crustacean sequenced genome. Thus, the value of a comparative genomics research from its related proteomes (i.e., insects) might be somewhat limited.

ProtoNet is a global automatic classification scheme for the entire protein space [6,7]. ProtoNet 6.0 provides a hierarchical organization of 10 million protein sequences [8]. The hierarchy results from an unsupervised clustering method that groups proteins according to their mutual similarity. The resulting hierarchy consists of protein clusters that are arranged into several trees. Each such tree represents a protein family at a different granularity - from a broad superfamily to a specialized subfamily [9]. Following pruning of the ProtoNet 6.0 family tree, the system reports on ~162,000 high quality stable clusters (for definitions, see Methods). ProtoNet was applied successfully as a complementary methodology for annotating newly sequenced genomes [10]. The incorporation of external annotation sources that cover structure, function, domain and taxonomy perspectives leads to impartial biological knowledge and functional inference [11,12].

In this study, we claim that the scaffold of ProtoNet can be successfully used for annotating the Daphnia full-length proteome. We show that by applying strict filters on the ProtoNet tree and adding a number of constrains for functional inference, we could safely map to preexisting clusters 98.7% of the Daphnia's proteome. For 87% of the mapped proteome, functional annotations were securely assigned. We show that the Daphnia proteins are clustered into ~8800 clusters, but only 40% of these clusters include insects' representatives. Most (61%) of the proteins are mapped to ~3000 clusters that contain at least 2 Daphnia's paralogs. We consider the function of the clusters that are exceptionally amplified relative to the fruitfly proteome and those that are maximally enriched in the Daphnia's proteome. We focus on ion channels and cuticle structural families that dominate the amplified duplicated genes. We discuss the relevance of gene expansions and the potential of the organisms to cope with the changing environment.

## Results

### Automatic mapping of the Daphnia proteome

**Figure 1:**
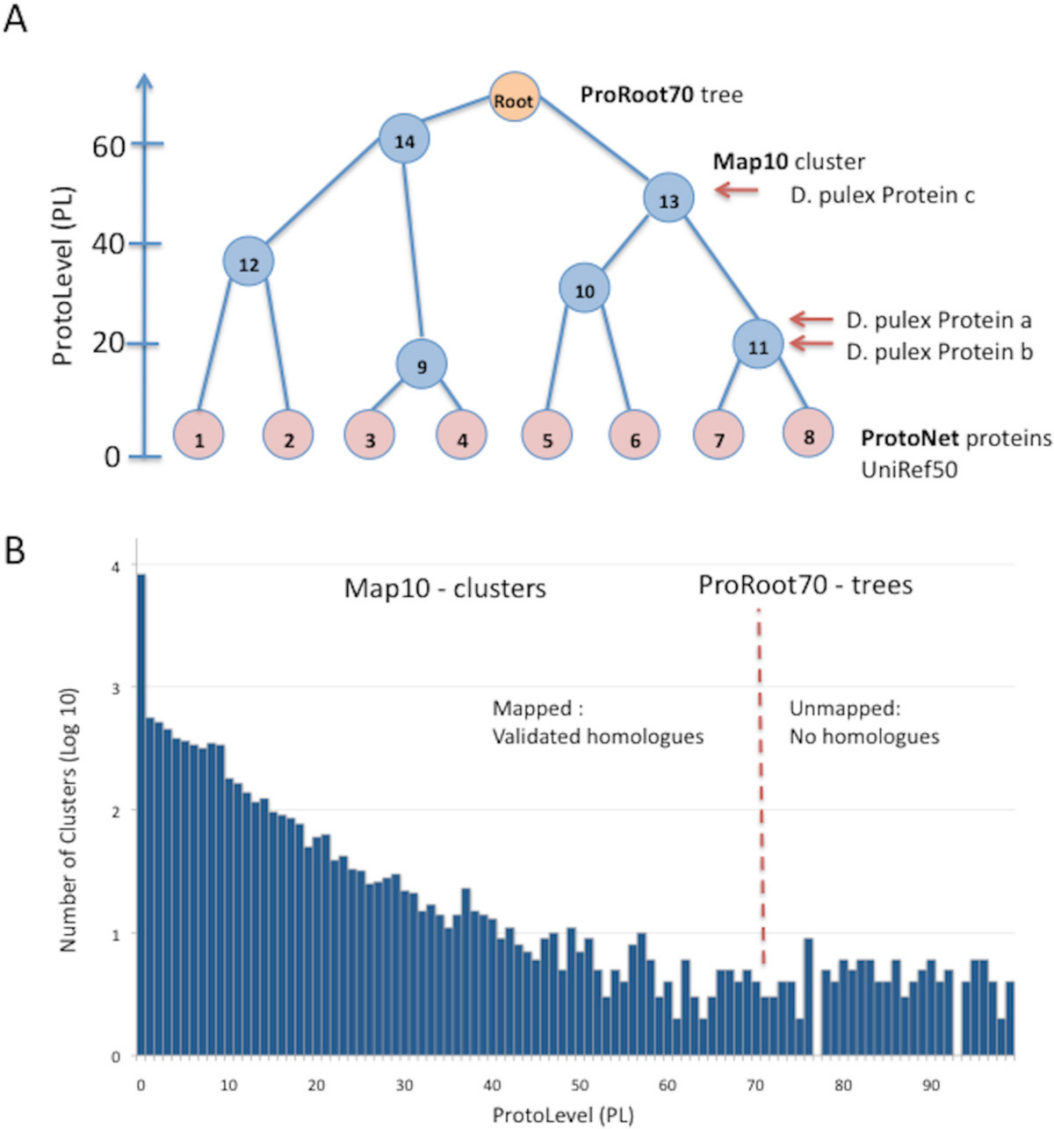
**(A) A scheme of the pruned ProtoNet tree**. The leaves of the tree (marked pink) are representatives of UniRef50. The left axis represents the ProtoLevel (PL) of the clusters. The lowest PL (PL = 0) is associated with the leaves. High PL (up to 100) is reached next to the global ProtoNet tree root. Each Daphnia protein (a-c) was mapped to 'best stable' node of the protein in the ProtoNet (Map10 clusters). The proteins that are mapped to the same node (e.g., node 11) are considered paralogs. Following mapping, we 'climb' the tree to a higher level of the hierarchy (PL = 70). The roots that contain Daphnia proteins are subjected to further analysis. Each ProRoot70 is conjectured to represent a functional family. **(B) **ProtoLevel of the mapped clusters. Only 129 clusters of Map10 are at PL> 70 (to the right of the red dashed line). These clusters are excluded from the annotation scheme.

The fully sequenced Daphnia pulex proteome comprises of 30,550 open reading frames (ORFs). We limited the analysis to 26,968 (88%) proteins that are full length. We mapped these proteins to the ProtoNet tree (see Methods) that was pruned to ensure high confidence clusters. Two parameters govern the validity of ProtoNet families (clusters): (i) the ProtoLevel (PL) that determines the depth of the tree. PL = 0 indicates the proteins as singletons and PL = 100 marks the ProtoNet root with the maximal number of merges at the root of the tree. (ii) The LifeTime (LT) is an intrinsic measure that approximates the stability of the clusters (see Methods). LT = 0 refer to a full representation of all clusters, i.e. a binary tree with the number of clusters that are identical to the number of protein within (> 9 millions [8]). LT = 1 is the default for semi-stable clusters. Towards the goal of mapping the Daphnia proteome to top confident clusters, we determined the LT (LT = 10, marked Map10, Figure 1A). Following mapping, we 'climb' the tree to a higher level of the hierarchy (PL = 70). The trimmed ProtoNet at PL = 70 is called ProRoot70 (Figure 1A). The pruned and compressed ProtoNet is used as the scaffold for the annotation task. Each ProRoot70 root is conjectured to represent a functional family.

**Figure 2:**
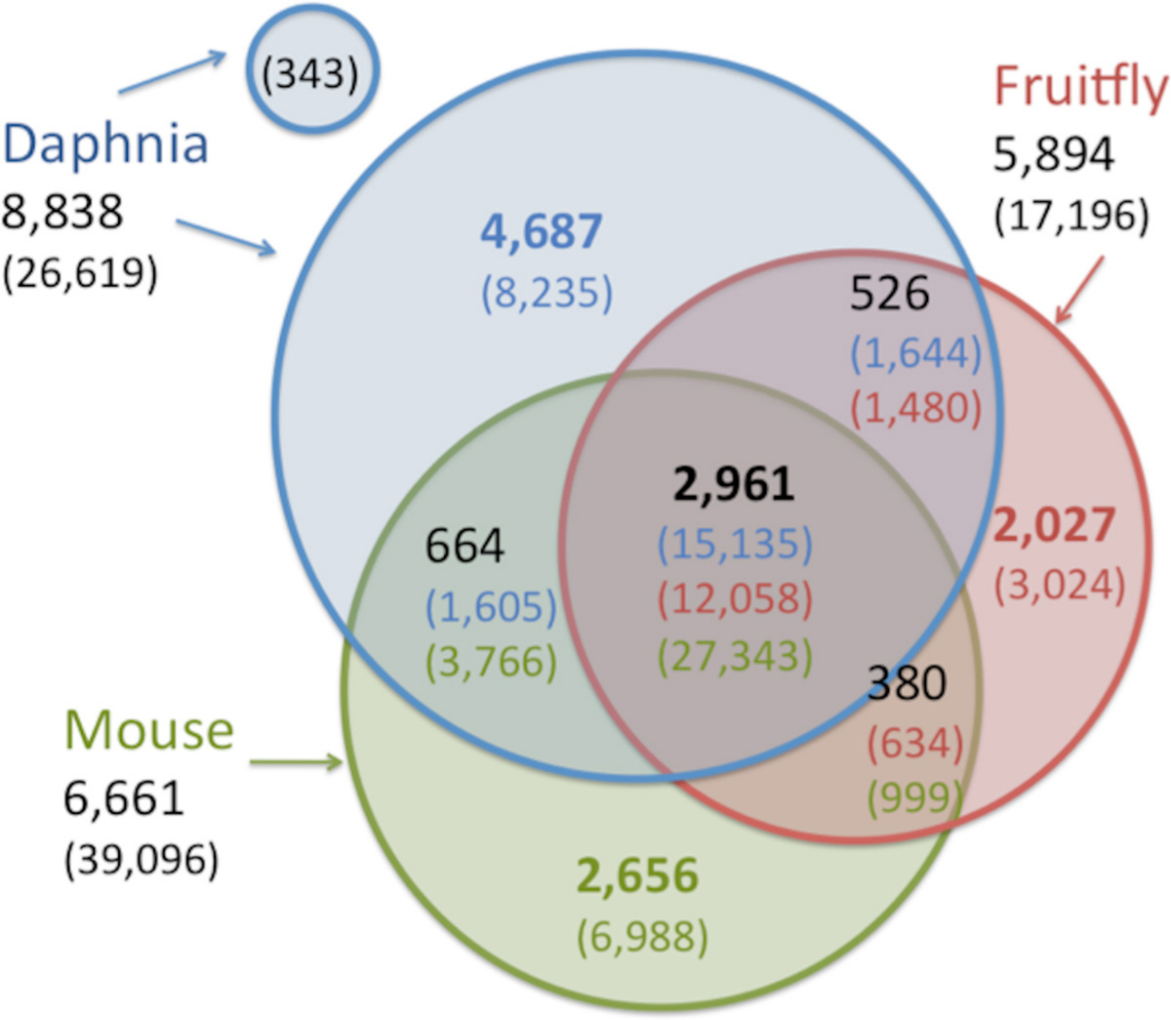
**A Venn diagram partitioning the ProRoot70 clusters for *Daphnia pulex, Drosophila melanogaster *and *Mus musculus *proteomes**. For each section in the diagram, the number of ProRoot70 clusters and the number of proteins for each of the analyzed organisms are indicated. For example, there are 664 ProRoot70 clusters with Daphnia proteins and mouse proteins, but with no Drosophila proteins. This section composes of 1,605 and 3,766 proteins, from the Daphnia and mouse respectively.

We mapped the Daphnia's proteins to: (i) the minimal-sized cluster from the ProtoNet 6.0 that met the merging criteria [13]; (ii) the predefined criteria of LT = 10. All together, we mapped 26,625 Daphnia's protein sequences to 13,880 clusters (i.e., Map10, the mapped clusters for the Daphnia proteome, Figure 1B). Only 343 proteins (1.3%) failed in their mapping. Figure 2B shows that the mapping of the Daphnia's proteins occurs at all levels of the tree, as indicated by the PL index. Among the 26,625 proteins, only 164 were mapped at PL> 70 (mapped to 130 clusters, Figure 1B). We will not discuss these proteins due to their questionable quality. In summary, less than 2% of the Daphnia full-length proteins failed our annotation scheme.

In order to achieve a global taxonomic view of the Daphnia proteome, we took two perspectives: (a) A protein-based view: Each of the 26,625 Daphnia sequences belongs to one of the ProRoot70 roots. Proteins assigned to the same root belong to the same functional family. For each protein, we check whether it has homologues from the mouse and the fruitfly (*Drosophila melanogaster*). (b) A root-based view: In ProRoot70, 8838 clusters contain at least one Daphnia's mapped protein. Among the ProRoot70 trees, 2953 clusters contain at least 2 Daphnia's proteins. For each ProRoot70, we check whether it contains proteins from the mouse, fruitfly or other organisms, in addition to the Daphnia proteins. The mouse and the fruitfly were selected as representatives for complex, 'complete proteomes'. In addition, these organisms differ considerably in their evolution history, mutation rate, generation time and other parameters that govern their protein families (see discussion in [14]).

We repeated the mapping protocol and thresholds as used for the Daphnia proteome for mapping the 17,438 and 39,386 full-length proteins from the fruitfly and the mouse, respectively. Figure 2 shows the results in a Venn diagram. As expected, a large majority (57%) of the proteins have homologues in the mouse and the fruitfly. Interestingly, a substantial fewer roots associate with the *D. melanogaster *proteome (5894 relative to 8838 ProRoot70 trees). About 40% of Daphnia's clusters include also proteins from the fruitfly. Notably, the fraction of proteins for [Daphnia+/Fruitfly+/Mouse-] or [Daphnia+/Fruitfly-/Mouse+] is identical, with 6% of the Daphnia proteome in each cross-taxa groups (Figure 2).

The proteome of the Daphnia includes many previously unseen proteins that have no homology to mouse or to the fruitfly (30%). Importantly, these 8235 proteins (Figure 2) are mapped to ProRoot70 that include other organisms. The number of proteins that are unique to the fruitfly or the mouse comprises 17% of their analyzed proteome (Figure 2). An interesting subset of proteins is the group of proteins that failed mapping (343). These proteins are potentially Daphnia specific proteins. However, these are prone to mistakes in genome annotations, and therefore, will not be further discussed.

### Automatic annotations of the Daphnia proteome

The principle underlying the assignment of annotations to the uncharacterized Daphnia proteome relies of the functional coherence in the ProRoot70 set. Previous quality assessment showed that the clusters of ProtoNet are of high quality in view of their annotations [8]. The sources for the automatic functional annotation task cover the standardized vocabulary of Gene Ontology (GO) (Camon et al. 2004, Harris et al. 2004), UniProt Keywords ([15], Pfam [16], Pfam, InterPro [17] and additional structural and functional classifications [18,19].

**Figure 3:**
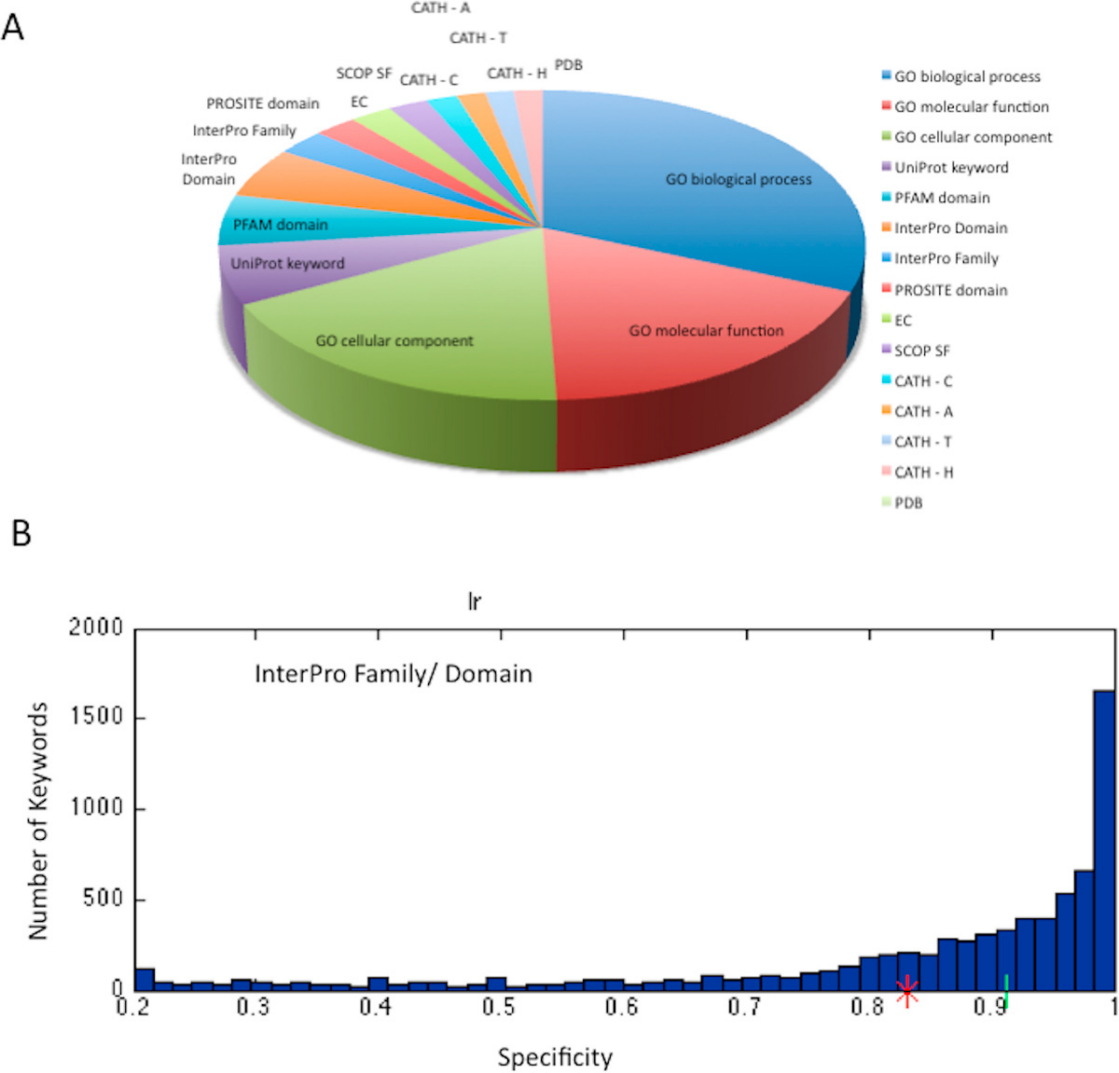
**Automatic functional annotation of Daphnia protoeme**. **(A) **The partition of the ProRoot70s' ProtoName sources. GO terms are dominating the annotations of the Daphnia proteome (67%). Each of the following resources, InterPro, Pfam and UniProt contributes an additional of 5-8% of the annotations. The rest of the annotations are from structural (SCOP, CATH) and functional classifications (Enzyme, EC). **(B) **Specificity (purity) score for the ProRoot70 is calculated in respect to InterPro annotations (Family and Domain). The average (0.84) is shown with a red asterisk. The median (0.9) is marked with a green line.

The partition of the resources that contribute to the successful Daphnia's proteome annotations task is shown (Figure 3). We use the concept of ProtoName for the annotations that best describe the cluster's proteins (see Methods). Recall that each cluster in ProtoNet is associated with many annotations. Thus, a representative cluster in ProtoNet will have a rich 'composed ProtoName'. We included filters for securing the confidence of the annotation inference process: (i) Specificity for the cluster is ≥ 0.2; (ii) The cluster size is ≥ 5 proteins. Using the filtration scheme, we were able to assign annotations for 73% of the proRoot70 (covering 86% of the Daphnia proteome) with an average of 13.7 annotations per proRoo70. Figure 3 shows the annotation sources according to all the terms used. Importantly, the annotation assignment is based on a fully automated procedure. The 3 branches of the GO terms dominate the annotations of the Daphnia proteome. Each of the sequence-based resources (Pfam, InterPro and UniProt) contributes additional 5-8% of annotations (Figure 3A).

We tested the quality of the ProRoot70 clusters that include Daphnia's proteins, using the specificity score (Figure 3B). The average specificity score for all InterPro terms (families and domains together) is 0.84 (the specificity median score is 0.9). This high specificity is a strong support for the quality of our automatic inference procedure.

The assignment of high quality annotations with a taxonomical view (Figure 2) allowed focusing on the functions that dominate the [Mouse+/Daphnia+/Fly-], and the complementary group [Mouse-/Daphnia+/Fly+]. Table 1 shows the sample of the largest ProRoot70 trees. We show that, the [Mouse+/Daphnia+/Fly-] families are rich with extracellular domains, proteases, heat shock proteins and calcium binding proteins. On the other hand, the [Mouse-/Daphnia+/Fly+] trees include representatives of the sensory apparatus (e.g., olfactory receptors, odorant binding proteins).

**Table 1:** The largest trees for [Daphnia+/Mouse+/Fly-] and [Daphnia+/Mouse-/Fly+].

Root ID ProRoot70	**# Daphnia-Mouse proteins**^a^	ProtoName ProRoot70	General term
4495737	1166	Olfactory receptor	Receptor
4480698	141	Immunoglobulin V-set	Binding-EX
4486320	42	NACHT nucleoside triphosphatase	Enzyme
4486050	33	S100/CaBP, calcium binding	Binding
4380861	31	Crystallin-fold	Fold
4385886	31	Hyaluronic acid binding	Binding-EX
4381234	29	2'-5'-oligoadenylate synthetase 1	Enzyme
4474452	27	Fibronectin type III	Binding-EX
4489975	25	Proteinase inhibitor I25, cystatin	Enzyme
4509859	18	Endoglin/CD105 antigen	Binding-EX

**Root ID** **ProRoot70**	**# Daphnia-Fruitfly proteins**^a^	**ProtoName ProRoot70**	**General term**

4490041	57	Insect cuticle protein	Structure
4493131	54	Olfactory receptor, Drosophila	Receptor
4434866	52	Insect cuticle protein	Structure
4476453	28	MADF domain/DNA binding	Binding
4392808	26	Protein of unknown function DUF243	
4310716	23	Odorant binding protein	Binding
4425478	16	Protein of unknown function DUF229	
4484179	14	Insect pheromone/Odorant binding	Binding
4511439	6	Trehalose/Gustatory receptor	Receptor
4351940	5	Metazoa	

^a^The number of proteins is the number in both organisms. EX- enriched in extracellular proteins.

### Most Daphnia's proteins have paralogs

**Figure 4:**
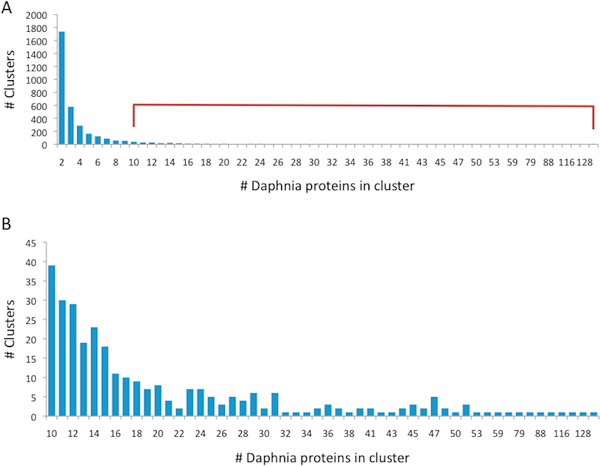
**The number of Daphnia's paralogs**. **(A) **At least two Daphnia's proteins are found in 24% (3,395/13,880) of the Map10 clusters. **(B) **There are 301 clusters with ≥ 10 paralogs and 98 clusters with > 20 paralogs.

Following mapping of the full-length proteome in 8838 clusters (ProRoot70), we found that 20,508 proteins (77%) were mapped to clusters that contain paralogs (i.e. contains at least 2 Daphnia's proteins) at the level of ProRoot70. We tested the paralogs at the level of Map10 (Figure 1). Notably, most of the Map10-clusters are at the granularity of families. These clusters often merge to bigger clusters that form families and superfamilies at the ProRoot70 level. About 24% of the Map10 clusters (16,134 proteins) include Daphnia's paralogs (Figure 4). Notably, there are 301 clusters with ≥ 10 paralogs and 98 clusters with > 20 paralogs (Figure 4B).

**Figure 5:**
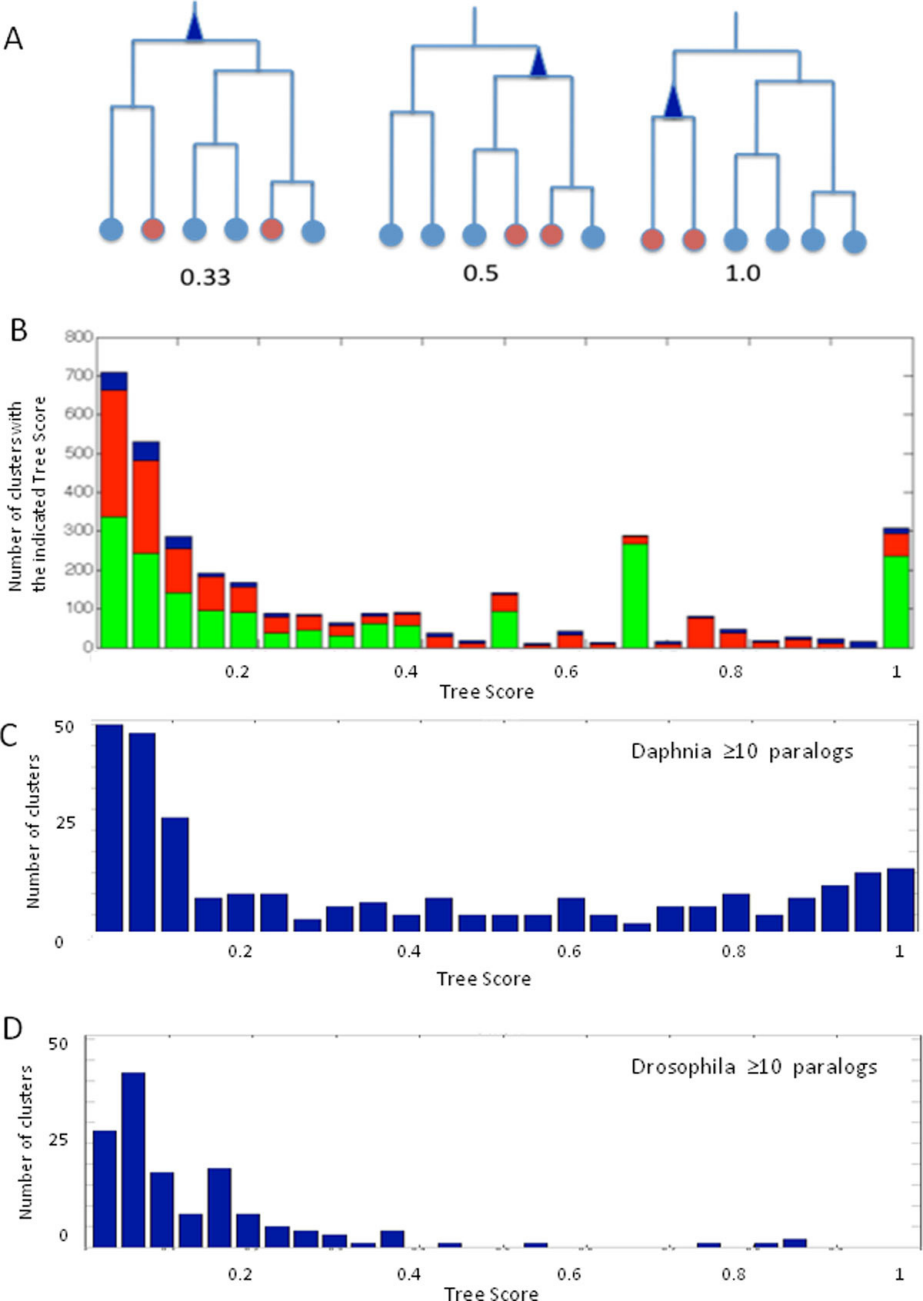
**The divergence of the Daphnia's paralogs**. **(A) **Tree Score (TS) is the number of proteins in the distance tree divided by the number of leaves in the minimum sub-tree which include all the proteins of interest. In the scheme, the minimum sub-tree that contains all of the protein of interest (marked red) is indicated with a blue triangle. If the proteins of interest diverged from each other, then the TS is small (left side of the graph). In the case for TS = 0.33, the proteins of interest are intermix with the other proteins. In the opposite case, the proteins of interest (marked red) are close to each other in the distances tree (right tree), so the sub-tree will have a maximal value of the TS (up to 1.0). **(B) **Histogram of the TS partitioned according to the number of paralogs. Clusters with two mapped Daphnia proteins are marked green. Clusters with 3-9 mapped Daphnia proteins are marked in red. Clusters with at ≥ 10 Daphnia mapped proteins are marked in blue. Histogram of TS of Daphnia **(C) **and Drosophila **(D) **for clusters with ≥ 10 mapped proteins. Note that Daphnia's TS values cover the entire range of TS (from 0 to 1.0) while high TS clusters are missing in Drosophila.

We tested the degree by which the Daphnia's proteins are separated or intermix with the other proteins at their Map10 cluster. The extreme case in which Daphnia proteins within the cluster remain as a separated sub-tree correlates well with a trend of low divergence. We tested the relation of Daphnia's proteins with respect to the other proteins in the mapped cluster using the Tree Score (TS, see Methods). Briefly, for each cluster that includes Daphnia's paralog at Map10 (Figure 4), we run BLAST in 'all against all' mode and create a distance binary tree (using ClustalW, [20]). For each tree, we computed the TS. It is simply the number of Daphnia proteins in the cluster proteins (leaves) divided by the size of the cluster (number of total leaves) of the minimum subtree that contain all the Daphnia proteins (Figure 5A). The TS ranges from 1.0 to a small positive value. When the lower common ancestor that combined all Daphnia's paralogs of the cluster is the root of the subtree (Figure 5A, left), the score is minimal.

Using the TS, we indirectly estimated the conservation relative to the size of the cluster subtree that contains all of the Daphnia's proteins within. We identified 305 clusters of TS = 1.0. High TS is indicative of the 'isolation' of the Daphnia's proteins from the other members in the cluster. 54% of the Daphnia's paralogs are associated with high divergence (TS < 0.2, Figure 5B). We examine the Map10 clusters that contain a large number of Daphnia's proteins (≥ 10). Such clusters are spread at all ranges of the TSs (Figure 5C). When the same analysis was performed on *Drosophila melanogaster *Map10 clusters, the dominating TSs are typically < 0.2, and no cases of high TSs were noted (Figure 5D). The results suggest that in Daphnia (but not the fruitfly), paralogs having low divergence in view of other proteins in the clusters are prevalent. A quantitative comparison of the paralogs in Drosophila and Daphnia was performed. The number of ProRoot70 roots that contain paralogs is 3029 and 2306 in Daphnia and Drosophila, respectively. The relation of the TS and the Tree size (i.e. number of leaves in the analyzed cluster) is shown for Daphnia (Additional file 1).

### Functional view on Daphnia's families with amplified paralogs

We inspected the annotations that are associated with clusters having a high number of duplicated genes (≥ 60 paralogs, Additional file 3). The results show that these clusters are rich with viral origin, apparently as relics of transposition events (e.g., integrase) [21]. Other such families include structural proteins of the cuticle and the cytoskeleton, large families of enzymes (e.g., protein kinase), and various signaling receptors (e.g., GPCR).

Table 2 shows the list of ProRoot70 trees with > 100 Daphnia's paralogs. Inspecting the ProtoNet clustering process provides an additional insight on their functional groups (Table 2). Specifically, the ratio between the number of paralogs in ProRoot70 cluster and the number of mapped clusters (Map10) along the hierarchy is informative (see Figure 1A). We focused on the clusters with a maximal number of paralogs (≥ 60, Additional file 3). We noted two extreme instances: (i) Roots of steadily growing proteins subfamilies (ratio < 10, Table 2). These clusters have known functions (e.g., Zn fingers, protein kinase) (ii) Roots that are composed of a small number of merges (ratio > 10, Table 1). Interestingly, ProRoot70 trees with such ratio (> 10) are typically associated with small clusters of a narrow taxonomical breath. Among these clusters are paralogs from viral origin and structural elements, mainly cuticle's components (Table 2, Additional file 3).

**Table 2:** Functional annotations for Daphnia's proteome at ProRoot70 (> 100 paralogs)

Root ID ProRoot70	# Daphnia proteins (ProRoot70)	# Daphnia proteins (Map10)	Ratio^a^	Functional group^b^	ProtoName (ProRoot70)
4510706	498	364	1.37	Enzyme	Protein kinase
4510983	279	155	1.80	Structure	ANK repeat
4507452	228	133	1.71	Interaction	WD repeat
4498845	228	104	2.19	Enzyme	Peptidase S1A
4508421	186	166	1.12	Interaction	Classic Zinc Finger
4490041	169	5	33.80	Structure	Insect cuticle protein
4506993	166	52	3.19	Viral	RNA-dep. DNA polymerase
4491232	160	8	20.00	Receptor	Glutamate receptor-related
4504048	155	80	1.94	Receptor	7TM GPCR, rhodopsin-like
4510005	140	33	4.24	Structure	Structural molecule activity
4502875	134	12	11.17	Interaction	Kelch related
4510835	128	92	1.39	Receptor	ABC transporter-like
4434866	123	3	41.00	Structure	Insect cuticle protein
4504753	123	10	12.30	Viral	DNA/RNA helicase
4450084	114	2	57.00	Viral	HpI Integrase; Chain A
4510417	108	62	1.74	Viral	Ribonuclease H-like
4510284	104	53	1.96	Interaction	Immunoglobulin-like
4372467	102	5	20.40	Viral	MULE transposase, domain
4508558	101	60	1.68	Interaction	RNA recognition motif, RNP-1

^a^Ratio, the ratio between the number of proteins and the number of mapped clusters. ^b^Viral, indicative of a transposome with a viral origin.

### A taxonomical imbalance of Daphnia paralogs

Based on the completeness of the Daphnia's genomes, we could focus on protein families that are characterized by a taxonomically imbalanced. Specifically, ProRoot70 trees that contain a high proportion of Daphnia:fly proteins may suggest gene amplifications that support essential function in Daphnia. In order to highlight taxonomically imbalanced clusters, we defined a taxonomical balance score (TB score, see Methods).

**Figure 6:**
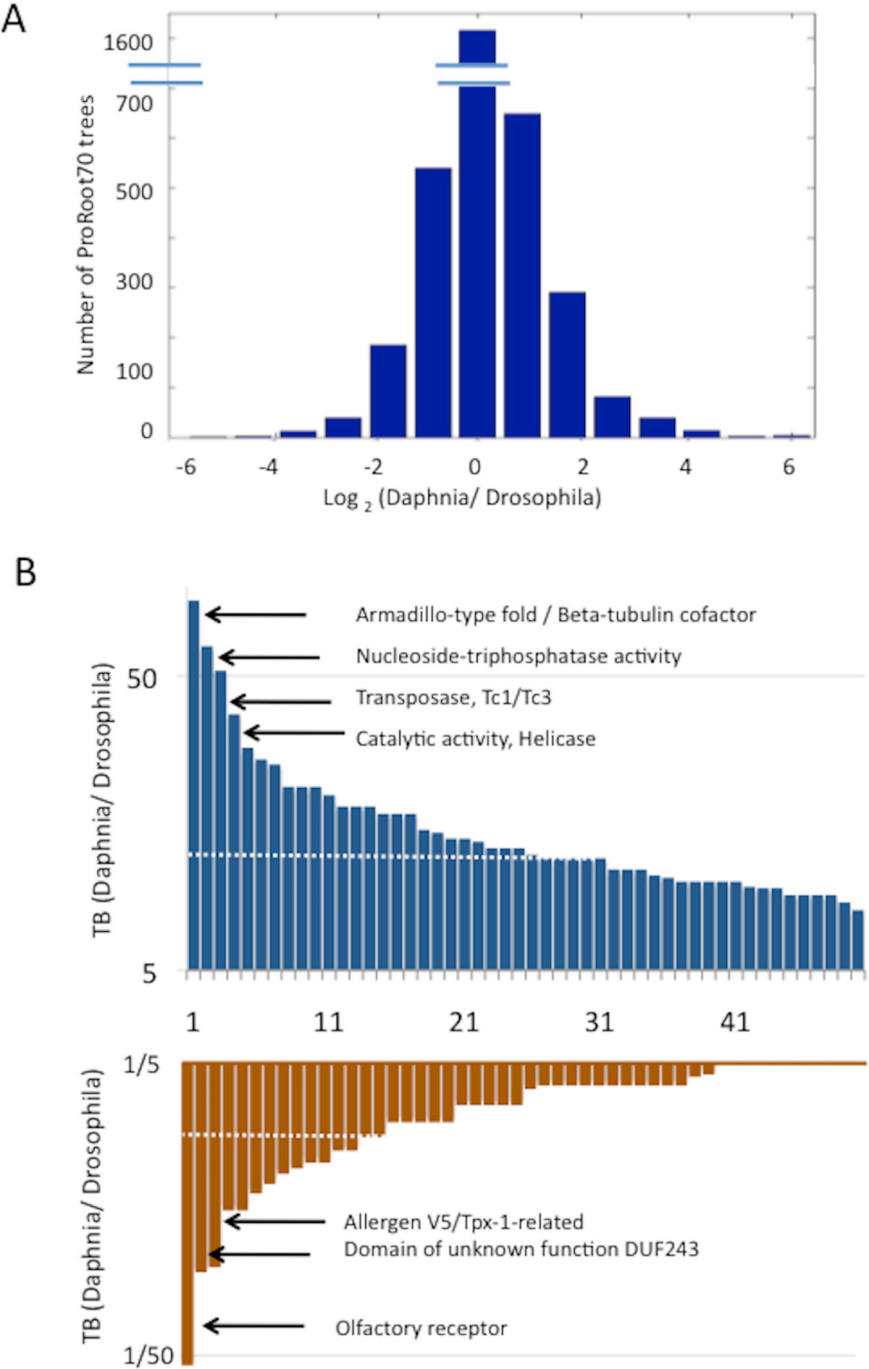
**Taxonomy Balance (TB) measures for Daphnia's clusters at ProRoot70**. **(A)**. Histogram is shown in log_2_(TB). For each proRoot70 tree that includes at least one Daphnia's and one Drosophila's protein, TB is computed as the number of Daphnia proteins divided by the number of Drosophila proteins. Note that the histogram is not symmetric. There are more ProRoot70 clusters with high numbers of proteins from Daphnia relative to Drosophila. **(B) **TB for 50 protein families with a maximal (or minimal) TB values. There are 31 clusters with TB ratio ≥ 10 and only 13 clusters with TB ratio ≤ 0.1 (i.e. > 10 folds more Drosophila's proteins relative to Daphnia's proteins, dashed lines). Annotations that are associates with selected clusters are indicated.

Figure 6A shows the TB score in log2 scale. The analysis was performed on ProRoot70 trees that contain the Daphnia's and the fruitfly proteins (3487 clusters, Figure 2). Most clusters have a TB = 0 indicating that there is no difference in the ratio of Daphnia and fruitfly proteins in the ProRoot70 trees.

Figure 6B shows the TB for the 50 protein families with a maximal (or minimal) TB values. There are 31 clusters with TB ratio ≥ 10 and only 13 clusters that have a TB ratio ≤ 0.1 (i.e. > 10 folds the number of Drosophila relative to Daphnia paralogs) (Figure 6B, dashed line). The functions associated with TB ratio ≥ 10 include nucleic acids regulation (Zn-fingers, HAT dimerization, ATPases), proteins of the stress response (Heat Shock, Clp1), Oxidative phosphorylation (Oxidoreductase, Cytochrome C) and transporters (Major facilitator, Lipid transport, ABC transporter). Drosophila paralogs with high TB ratio (≥ 10) confined to clusters of unknown functions, pheromone and olfactory receptors (Figure 6B).

The TB test indicates the relevance of this measure to the behavior and the environmental difference between the fruitfly and the Daphnia. For example, the essential requirements for stress response elements in Daphnia are exposed through the Dapnia:fly TB score.

### Manual evaluation: plasma membrane receptors and ion channels

Inspecting the ProRoot70 trees that contain a large number of Daphnia's proteins revealed families that are particularly enriched with receptors and signaling proteins. We consider three such families that are characterized by a high ratio of the number of paralogs (in the ProRoot70) relative to Map10 clusters (Table 1) and a high TB value relative to the fly (Figure 6). We focus on the amplifications of ion channels and receptors.

The assignment of a large group of Daphnia's paralogs to the ionotropic glutamate receptors is intriguing. Daphnia's representatives were found for each of the three subclasses of glutamate receptor (ProRoot70, ID 4491232): (i) The NMDA (N-methyl-D-aspartate) receptors are highly permeable for Ca^2+ ^ions. NMDA receptors play a key role in the plasticity of the nervous system. (ii) The AMPA (alpha-amino-3-hydroxy-5-methyl-4-isoxazole-4-propionic acid) receptors that are the most commonly found receptors in the nervous system, and (iii) the Kainate receptors.

ProRoot70 tree with a ProtoName of 'Ionotropic glutamate receptor' (InterPro) includes 160 of the Daphnia's paralogs. The InterPro term covers 140/160 instances. The surprisingly high prevalence of glutamate receptors (AMPA, Kainate and NMDA) is most likely to control the excitatory synaptic transmission in the crustacean brain. Specifically, transient activation of NMDA receptors leads to a modification in the strength of synaptic transmission mediated by AMPA and kainate receptors. The ionotropic glutamate receptors are ancient in evolution. Events of genes loss and gain were reported for these genes along the phylogenetic tree [22]. A collection of 160 related gene products in Daphnia has the potential for a rich combinatorial array of ion channels and sensors.

Another functional group includes the Daphnia's paralogs identified as Bestrophin. The Bestrophin is a family of plasma membrane proteins that express in the retinal pigment epithelial cells. Mutations in the homologous human gene cause 'BEST Macular Dystrophy' disease. Bestrophins compose a new class of chloride channels that are restricted to multicellular metazoa. Daphnia's paralogs mapped to the largest Bestrophin subfamily (54 proteins, based on PANTHER [23]). In this subfamily, the other proteins are from the fruitfly (4 proteins) and Caenorhabditis (C. briggsae and C. elegans with 21 and 25 proteins, respectively).

A remarkable amplification is detected for the 51 Daphnia's proteins that are mapped to Ryanodine receptors (RyR) and inositol 1,4,5-trisphosphate receptors (IP3R) ProtoNet family. These proteins belong to the superfamily of ligand-gated intracellular Ca^2+ ^channels. The RyR and IP3R control the Ca2+ homeostasis of the cells and are essential in neurons, muscle and other secreting cells. The IP3 receptor acts as a Ca2+ release channel from internal stores in smooth muscle and non-muscle tissues. However, at high Ca^2+ ^concentrations in the cytosol, IP3 receptors are inhibited. Such inhibition is an essential mechanism for terminating the channel activity and thus preventing pathological Ca^2+ ^rises.

**Figure 7:**
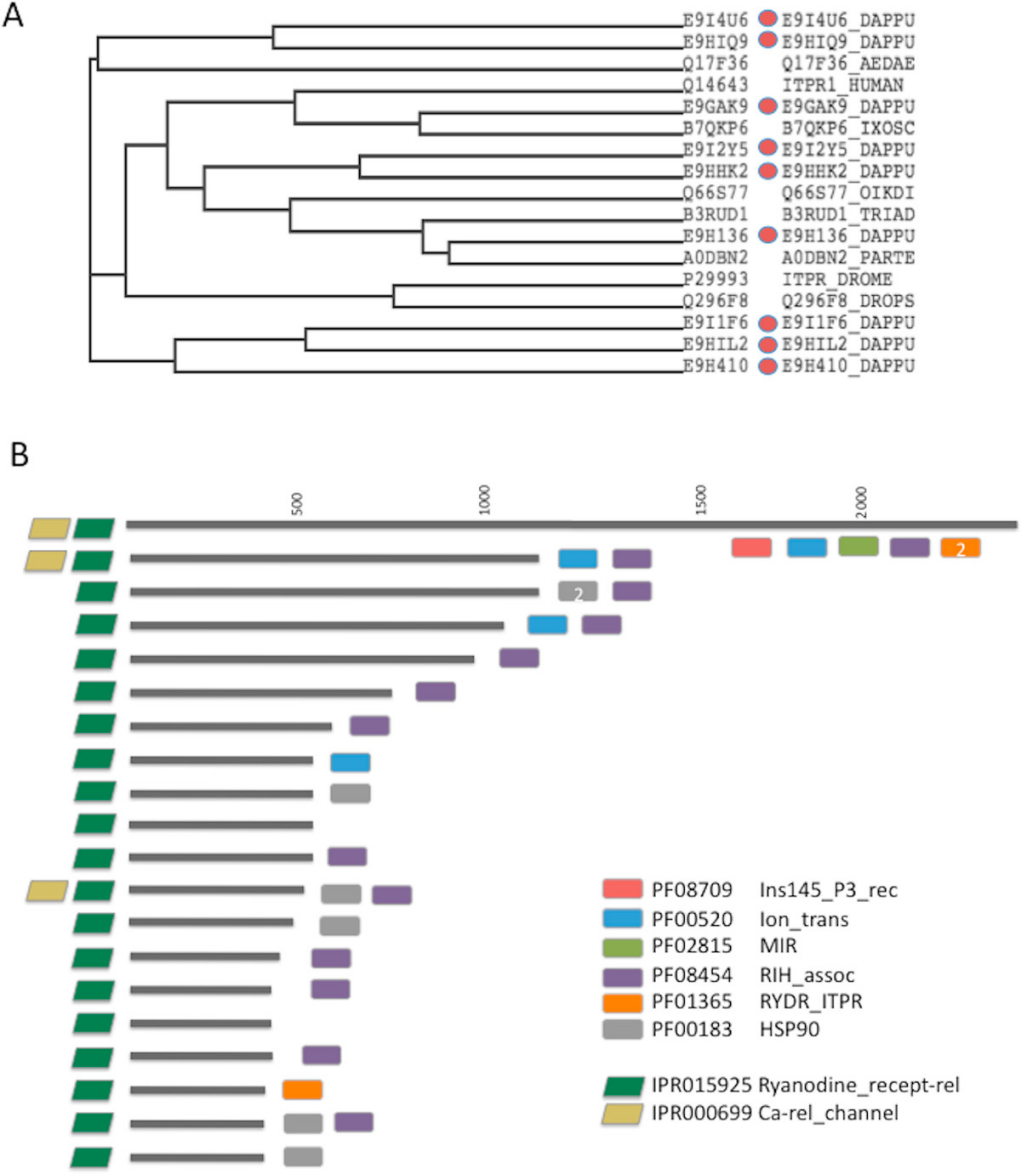
**Daphnia's paralogs of Ryanodine and IP3 receptor families**. **(A) **Dendogram based on multiple sequence alignment (MSA) of IP3 receptors including the Daphnia protein E9HHK2. The Daphnia proteins (red symbol) intermix with IP3 receptor proteins from other organisms. **(B) **A schematic view of the collection of Ryanodine like receptors according to their domain architectures and based on InterPro (Ryanodine receptors and Ca^2+ ^release channel). The domains according to Pfam are also listed (rectangle symbols). Interestingly, most Daphnia's proteins in the cluster are shorter than the average length of the Ryanodine and IP3 receptors. Only 8/51 Daphnia paralogs failed to meet the InterPro definition of 'Ryanodine related receptor'. Notably, the analyzed ProRoot70 ID 4478501 (65 proteins) contains proteins from human, fruitfly, unicellular ciliate protozoa and Paramecium. The Daphnia proteins presented are those with a minimal length of 350 amino acids.

The overwhelming number of Daphnia's proteins (51 proteins, ID 4200503) is restricted to the domains that characterize these receptors. The average length of the cluster is 2404 ± 927 amino acids. However, the length of the 51 Daphnia paralogs is only 352 ± 396 amino acids. Phylogenetic tree based on a multiple sequence alignment (MSA) of Daphnia protein E9HHK2 is shown (Figure 7A). Note that the Daphnia proteins are intermixed with IP3 receptors from other organisms including the Drosophila, Ades (mosquito), Trichoplax, Ixodes (tick) and more (Figure 7A). For the MSA see Additional file 2.

Figure 7B illustrates the collection of the Daphnia proteins (length > 350 amino acids) according to their domains and descriptors of InterPro as Ryanodine receptors and Ca^2+ ^release channel. The domains according to Pfam are listed (Figure 7B). Interestingly, despite the short Daphnia's proteins in the cluster, only 8 of the 51 Daphnia paralogs failed to meet InterPro definition of 'Ryanodine related receptors'. Notably, the ProRoot70 ID 4478501 (65 proteins) contains proteins from a broad collection of species including human, fruitfly, unicellular ciliate protozoa and Paramecium.

## Discussion

Most methods for functional inference are biased towards the detection of the 'known space' and fail in detecting novel families. A unique aspect of the ProtoNet method is the fact that it is unsupervised. We mapped the Daphnia uncharacterized proteome to ProtoNet 6.0. Once a new genome is sequenced, there are several tasks that may be performed with the goal of functional assignment. These approaches include (i) alignment-based comparative genomics; (ii) matching to predetermined statistical models (e.g. InterProScan). Domain and family-based resources provide an excellent coverage of the 'known space' using HMMs (12,000 in Pfam [24], 37,000 in EVEREST [25]). Iterative search using PSSM and HMM Profiles are often used for a comprehensive functional inference. However, all these methods consider each protein as a separate entity. Thus, a global perspective of the analyzed proteome is lost.

A growing number of proteomes, many of them are isolated in the species tree, become available. In the current study, intrinsic features of the data (e.g., PL and LT, Figure 1) guide the functional assignment. Specifically, the composed ProtoName captures the most significant annotations (Figure 3). ProtoName is linked to the majority of the stable clusters [8]. We suggest that our annotation process, in conjunction with supervised methods will provide a maximal coverage. ProtoNet 6.0 serves as the scaffold for the Daphnia annotation. The DB including all the external expert annotations (e.g., SCOP, Pfam, GO) will be updated each year. It will be beneficial to retest the performance sensitivity of inference following an update for all these resources. It will serve to assess the functional inference quality in view of the gradual improvement in external knowledge.

A similar approach, called ProtoBee, was applied for annotating the honey bee proteome [10]. ProtoBee tree was constructed from about 200,000 proteins including 10,000 proteins from the honey bee. About 70% of the bee's proteins were successfully annotated in this task [10]. Our current strategy for annotation assignment is based on mapping the 30,000 Daphnia's proteins on a scaffold of ProtoNet 6.0 tree-like structure. Almost 10 million proteins are included in such a family tree. The success in annotating the Daphnia proteome covers 86% of the full-length proteome, despite the high percentage of proteins that lack known homologues. The enhanced performance in annotating the Daphnia proteome stems from the use of 10 millions sequences from all domains of life. Furthermore, the number of external annotations such as InterPro and GO terms was almost doubled in the 5 years from the ProtoBee project [10]. We conclude that the drastic increase in data improved the performance of genome size automatic annotations.

In this study, we applied a taxonomical view to identify the unique clusters of crustaceans. In this view, [fly+/Daphnia-] and [fly-/Daphnia+] clusters are of a special interest (Figure 2). These sets account for functions that were lost/gain after the separation of crustaceans from insects. The taxonomical view provides an insight on genes that fulfill the Daphnia's unique needs. Evidence from other related genomes will be needed to substantiate the trends of gene loss and gain in crustaceans.

A large fraction of the Daphnis's proteome includes amplified genes. Instead of searching the proteins that meet an artificial predetermined threshold (e.g., Blast E-score < e-20), we mapped proteins to their most reliable cluster (Map10, Figure 1A) and followed their merges along the tree hierarchy. We identified that a fraction of the Daphnia's paralogs is characterized by a low divergence (Figures 5C, high TS). These paralogs are not mixed with other proteins in the cluster. However, such property was not detected among Drosophila's paralogs (Figures 5D). We assume that the Daphnia's paralogs that have high TB score reflect the dynamics of the Daphnia genome. The prevalence of proteins related to viral infection and transposition supports our hypothesis.

We determine hundreds of Daphnia's paralogs (Figure 4). It was noted that Daphnia pulex's genome appears to have twice as many gene duplication events with respect to the duplicate-rich *C. elegans *genome [26]. Gene duplication in *C. elegans *occurred more frequently than in Drosophila or yeast. Analysis for gene duplications in Ryanodine receptors (RyR) and IP3R (Figure 7) indicates that RyR and IP3R are spread in small groups of 2-5 genes at a chromosomal proximity. Such organization applies to many of the Daphnia's paralogs [2].

The TB score is designed to track the extreme instances of imbalance in the number of Daphnia's paralogs. We used the D. melanogaster as a reference for a model organism whose annotation is supported by experimental evidence. The striking enrichment in Daphnia's proteins, using the TB measure, includes cuticle structural elements (Additional file 3), transposon proteins and various ion channels (e.g., glutamate and RyR and IP3 receptors, Figure 7). Analysis of the chemoreceptors [22,27] suggests that the ionic glutamate receptors belong to a fast evolving superfamily. Similar observations for expanded gene families were reported for Daphnia ABC transporters [28], transposon proteins [21] and the Cytochrome P450 [29]. It is anticipated that a network of sensing and signaling molecules is essential for Daphnia's environmental response and acclimation against environmental toxicity.

## Conclusions

In this paper, we present a novel method that combines both the tasks of comparative analysis and automatic annotation. One unique aspect of the clustering method used is the fact that it is an unsupervised method. The protocol presented is useful in the annotation task of further genomes, especially in the case that there are no other related genomes in the public domain.

The uncharacterized Daphnia's proteome was mapped successfully to thousands of protein families. For 81% of these families, the functional inference from various external resources was successful.

An unbalanced taxonomical outlook for Daphnia proteome in view of the fruitfly as a model organism was instrumental to identify genes' amplification in Daphnia. These expanded protein families may underlie the capacity of Daphnia to cope with the environmental toxicity, oxygen availability, wide temperature range and other harsh conditions.

## Methods

### Protein clustering

All *Daphnia pulex *proteins that are not assigned as 'fragments' were extracted from UniProtKB (release of April 2011). All Drosophila and Mouse proteins were downloaded from UniProtKB and restricted to 'Complete Proteome' set. The organization of the proteins into a set of families is based on the scaffold of the ProtoNet 6.0 hierarchical tree [8] that includes 10 million proteins from UniProtKB [30].

The ProtoNet tree construction is described in [7,8]. The main steps in the hierarchical tree are (i) All-against-all BLAST. NCBI BLAST is run on all pairs of proteins, using BLOSUM62. All E-values lower than 100 are kept in a matrix. The E-values which are less significant than the value 100 are considered 100; (ii) Hierarchical clustering. An agglomerative clustering procedure is applied in which all clusters start as singletons, and at each step the two clusters that have the lowest score are merged into a new cluster. The score between two clusters is defined as the arithmetic mean of the E-values from all inter-cluster pairs of proteins. An efficient clustering algorithm was implemented [31]; (iii) Stable cluster and pruning. We only consider clusters that are stable. To this end, we chose Life Time (LT) = 10 for mapping the Daphnia proteins to a subset of robust clusters (Map10); (iv) ProtoLevel 70 was selected for defining the root clusters. The proteins of each of the Map10s are contained in its root cluster of ProRoot70. Therefore, the terms 'tree' and 'root' will be used interchangeably.

ProtoNet scaffold tree is used for classifying each one of the Daphnia's proteins according to the match with the best stable cluster. The Daphnia's clusters from the initial mapping are named Map10 clusters. The depth of the tress (ProtoLevel, PL) is used for estimating the relatedness of the sequences and the clusters' quality. ProtoNet has been shown to produce hierarchies for thousands of highly coherent clusters at high quality at PL that is > 90. We restricted the analysis to clusters' size that are limited by the PL = 70 to ensure the high confidence annotation inference. The collection referred to as ProRoot70 composed of 251,403 roots.

### Annotation inference

We focused only on the following dominating annotations: UniProt Keywords, EC, GO, InterPro and the structural classifications from CATH [32] and SCOP [33] (see database description in [8]). For each one of these keywords we looked for the one with the highest Correspondence Score (CS) index that reflects the size of the intersection (number of proteins with a specific annotation in the cluster) divided by the size of the union (number of proteins with the specific annotation in the tree). We eliminate annotations that are based on uninformative terms such as 'complete proteome', 'taxonomy' and 'hypothetical protein'.

Each mapped Daphnia protein is assigned the annotations that were given to the cluster to which it belongs and the annotations that were assigned to all the cluster's parents in the ProRoot70. Validated annotations were restricted to clusters that have at least 5 proteins and the cluster specificity is ≥ 0.2. The additional filtrations ensure the safe inference for 86% of the mapped Daphnia's proteome.

### Paralog definition

We marked *Daphnia pulex *proteins as paralogs for proteins that were mapped to the same Map10 clusters. Clusters that include at least two proteins from the subjected organism are called paralogs. There are 3395 clusters that contain paralogs (16,134 proteins). At the level of ProRoot70, there are 3029 such clusters. About half of them (1464) include more than one Map10 cluster.

### Additional scores

#### Tree Score

We used the Tree Score (TS) as an indirect measure for the separation of the Daphnia's protein in the Map10 clusters. For each cluster, a multiple sequence alignment was done for all proteins in the cluster and for the mapped proteins from the tested specie (*Daphnia pulex *or *Drosophila melanogaster*) using ClustalW, [20] with default parameters. Then, a tree was constructed according to the distance matrix. For each such distance tree, TS was computed. TS of a cluster is considered as the number of proteins of interest that were mapped to the cluster divided by the number of leaves in the smallest subtree containing all of them. Or in a formal notation: let T be a distances tree, $D_{T}$ is the set of leaves in T that belongs to the species of interest. And let $\Gamma_{T}$ be the set of all subtrees of T.

$$TS\left(T\right)=\frac{\left|D_{T}\right|}{\underset{\begin{array}{c} T_{i}\in \Gamma_{T}\\ s.t.\forall d\in D_{T},\;d\in T_{i} \end{array}}{\min}\left|T_{i}\right|}$$

Tree Score's range from 0 to 1.0. The relation between the TB and the size of the cluster is shown (Additional file 1).

#### Taxonomy Balance

The Taxonomy Balance (TB) index measures the imbalance between proteomes. It is measured as the ratio of the Daphnia proteins to any selected reference proteome (Drosophila, mouse) in a ProRoot70 cluster. Only ProRoot70 trees that contain at least one protein from each of the discussed proteomes are considered.

## Competing interests

The authors declare that they have no competing interests.

## Authors' contributions

NR processed the Daphnia genomic and proteomics data. NR and ML designed the experiments, evaluated the results, assessed the statistical significance and wrote the manuscript.

## Supplementary Material

Additional file 1 **Histogram of the TS partitioned according to the number of paralogs**. Click here for file

Additional file 2 **Taxonomical tree IP3R related proteins from *Daphnia pulex***. Click here for file

Additional file 3 **List of the ProRoot70 tress with ≥ 60 *Daphnia pulex *paralogs**. Click here for file

## Declarations

This article has been published as part of *BMC Bioinformatics *Volume 14 Supplement 3, 2013: Proceedings of Automated Function Prediction SIG 2011 featuring the CAFA Challenge: Critical Assessment of Function Annotations. The full contents of the supplement are available online at http://www.biomedcentral.com/bmcbioinformatics/supplements/14/S3.
