# Supplementary figures and images for: Functional inference by ProtoNet family tree: the uncharacterized proteome of Daphnia pulex
Source: BMC Bioinformatics. 2013 Feb 28;14(Suppl 3):S11. doi: 10.1186/1471-2105-14-S3-S11 (PMC3584848; doi:10.1186/1471-2105-14-S3-S11)

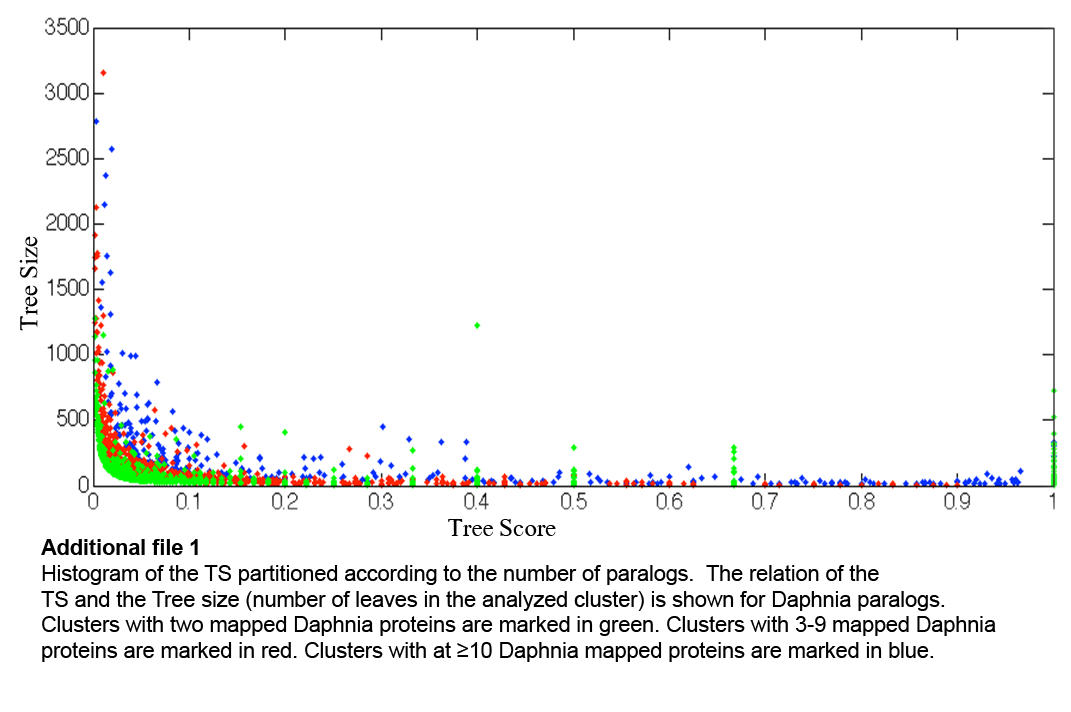

Supplement: Additional file 1 — Histogram of the TS partitioned according to the number of paralogs. [file 1471-2105-14-S3-S11-S1.TIF]

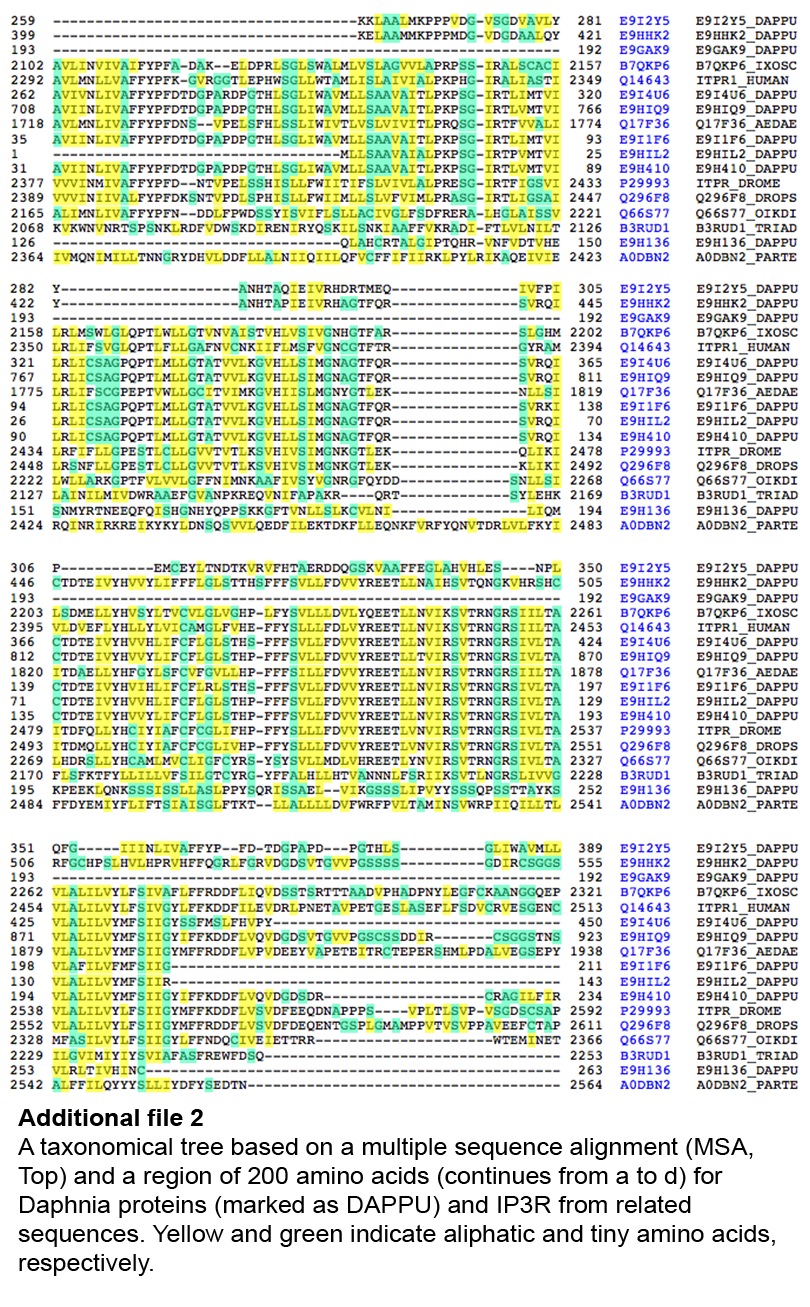

Supplement: Additional file 2 — Taxonomical tree IP3R related proteins from Daphnia pulex. [file 1471-2105-14-S3-S11-S2.TIF]
