# Supplementary material for: Functional inference by ProtoNet family tree: the uncharacterized proteome of Daphnia pulex
Source: BMC Bioinformatics. 2013 Feb 28;14(Suppl 3):S11. doi: 10.1186/1471-2105-14-S3-S11 (PMC3584848; doi:10.1186/1471-2105-14-S3-S11)
Supplement: Additional file 3 — List of the ProRoot70 tress with ≥ 60 Daphnia pulex paralogs. [file 1471-2105-14-S3-S11-S3.DOCX]

**Additional file 3.** ProRoot70 tress with ≥60 *Daphnia pulex* paralogs.

| Cluster ID ProRoot70 | # Daphnia clusters (Map10) | # Daphnia proteins (ProRoot70) | # Proteins/ # of mapped clusters | ProtoName (ProRoot70) | # Proteins (UniRef50) |
| --- | --- | --- | --- | --- | --- |
| 4510706 | 364 | 498 | 1.37 | Protein kinase | 15563 |
| 4510983 | 155 | 279 | 1.80 | ANK repeat | 5357 |
| 4507452 | 133 | 228 | 1.71 | WD repeat | 4795 |
| 4498845 | 104 | 228 | 2.19 | Peptidase S1A, chymotrypsin | 2327 |
| 4508421 | 166 | 186 | 1.12 | Classic Zinc Finger | 4956 |
| 4490041 | 5 | 169 | 33.80 | Insect cuticle protein | 337 |
| 4506993 | 52 | 166 | 3.19 | RNA-dependent DNA polymerase | 1909 |
| 4491232 | 8 | 160 | 20.00 | Glutamate receptor-related | 396 |
| 4504048 | 80 | 155 | 1.94 | 7TM GPCR, rhodopsin-like | 2690 |
| 4510005 | 33 | 140 | 4.24 | Structural molecule activity | 971 |
| 4502875 | 12 | 134 | 11.17 | Kelch related | 381 |
| 4510835 | 92 | 128 | 1.39 | ABC transporter-like | 16724 |
| 4504753 | 10 | 123 | 12.30 | DNA/RNA helicase, DEAD/DEAH | 2708 |
| 4434866 | 3 | 123 | 41.00 | Insect cuticle protein | 229 |
| 4450084 | 2 | 114 | 57.00 | HpI Integrase; Chain A | 24 |
| 4510417 | 62 | 108 | 1.74 | Ribonuclease H-like | 1788 |
| 4510284 | 53 | 104 | 1.96 | Immunoglobulin-like | 1416 |
| 4372467 | 5 | 102 | 20.40 | MULE transposase, domain | 34 |
| 4508558 | 60 | 101 | 1.68 | RNA recognition motif, RNP-1 | 2710 |
| 4499015 | 52 | 91 | 1.75 | Protein binding | 3024 |
| 4488038 | 2 | 90 | 45.00 | Armadillo-type fold | 70 |
| 4508125 | 33 | 85 | 2.58 | Cytochrome P450, C-terminal | 4398 |
| 4496270 | 4 | 84 | 21.00 | Reverse transcriptase, related | 275 |
| 4510121 | 68 | 78 | 1.15 | Histidine kinase | 14021 |
| 4498735 | 58 | 75 | 1.29 | SNF2-related, Helicase | 1936 |
| 4511563 | 46 | 75 | 1.63 | Glucose/ribitol dehydrogenase | 7902 |
| 4507020 | 48 | 72 | 1.50 | AMP-dependent synthetase ligase | 4802 |
| 4509968 | 56 | 71 | 1.27 | Ras | 1637 |
| 4465502 | 9 | 68 | 7.56 | Invertebrate chitin-binding | 345 |
| 4502589 | 12 | 65 | 5.42 | Eukaryota | 98 |
| 4511295 | 9 | 65 | 7.22 | Smc hinge domain | 1385 |
| 4506862 | 5 | 63 | 12.60 | Nucleoside-triphosphatase activity | 344 |
| 4511168 | 22 | 62 | 2.82 | Zinc finger, C3HC4 RING-type | 2234 |
| 4511024 | 50 | 61 | 1.22 | Kinesin | 1176 |
| 4499824 | 43 | 60 | 1.40 | Homeobox | 2116 |
| 4482823 | 10 | 60 | 6.00 | Ubiquitin thiolesterase | 1184 |
| 4482273 | 9 | 60 | 6.67 | Protein binding | 946 |
| 4470165 | 2 | 60 | 30.00 | Metazoa | 13 |
